# Peripheral administration of human recombinant ApoJ/clusterin modulates brain beta-amyloid levels in APP23 mice

**DOI:** 10.1186/s13195-019-0498-8

**Published:** 2019-05-10

**Authors:** Sofía Fernández de Retana, Paula Marazuela, Montse Solé, Guillem Colell, Anna Bonaterra, Jose Luis Sánchez-Quesada, Joan Montaner, Daniel Maspoch, Mary Cano-Sarabia, Mar Hernández-Guillamon

**Affiliations:** 1 Neurovascular Research Laboratory, Vall d’Hebron Research Institute, Universitat Autònoma de Barcelona, 08035 Barcelona, Spain; 2 Cardiovascular Biochemistry Group, Research Institute of the Hospital de Sant Pau (IIB Sant Pau), Barcelona, Spain; 3 CIBER of Diabetes and Metabolism (CIBERDEM), ISCIII, Madrid, Spain; 4 Catalan Institute of Nanoscience and Nanotechnology (ICN2), CSIC and the Barcelona Institute of Science and Technology, , Campus UAB, Bellaterra, Barcelona, Spain; 5 Institució Catalana de Recerca i Estudis Avançats (ICREA), 08100 Barcelona, Spain

**Keywords:** Clusterin, Apolipoprotein J, ApoJ, APP23, Reconstituted HDL, Alzheimer’s disease

## Abstract

**Background:**

ApoJ/clusterin is a multifunctional protein highly expressed in the brain. The implication of ApoJ in β-amyloid (Aβ) fibrillization and clearance in the context of Alzheimer’s disease has been widely studied, although the source and concentration of ApoJ that promotes or inhibits Aβ cerebral accumulation is not clear yet. ApoJ is abundant in plasma and approximately 20% can appear bound to HDL-particles. In this regard, the impact of plasmatic ApoJ and its lipidation status on cerebral β-amyloidosis is still not known. Hence, our main objective was to study the effect of a peripheral increase of free ApoJ or reconstituted HDL particles containing ApoJ in an experimental model of cerebral β-amyloidosis.

**Methods:**

Fourteen-month-old APP23 transgenic mice were subjected to subchronic intravenous treatment with rHDL-rApoJ nanodiscs or free rApoJ for 1 month. Aβ concentration and distribution in the brain, as well as Aβ levels in plasma and CSF, were determined after treatments. Other features associated to AD pathology, such as neuronal loss and neuroinflammation, were also evaluated.

**Results:**

Both ApoJ-based treatments prevented the Aβ accumulation in cerebral arteries and induced a decrease in total brain insoluble Aβ_42_ levels. The peripheral treatment with rApoJ also induced an increase in the Aβ_40_ levels in CSF, whereas the concentration remained unaltered in plasma. At all the endpoints studied, the lipidation of rApoJ did not enhance the protective properties of free rApoJ. The effects obtained after subchronic treatment with free rApoJ were accompanied by a reduction in hippocampal neuronal loss and an enhancement of the expression of a phagocytic marker in microglial cells surrounding Aβ deposits. Finally, despite the activation of this phagocytic phenotype, treatments did not induce a global neuroinflammatory status. In fact, free rApoJ treatment was able to reduce the levels of interleukin-17 (IL17) and keratinocyte chemoattractant (KC) chemokine in the brain.

**Conclusions:**

Our results demonstrate that an increase in circulating human rApoJ induces a reduction of insoluble Aβ and CAA load in the brain of APP23 mice. Thus, our study suggests that peripheral interventions, based on treatments with multifunctional physiological chaperones, offer therapeutic opportunities to regulate the cerebral Aβ load.

**Electronic supplementary material:**

The online version of this article (10.1186/s13195-019-0498-8) contains supplementary material, which is available to authorized users.

## Background

Cerebral β-amyloidosis is characterized by the accumulation of β-amyloid (Aβ) protein in the brain parenchyma and in cerebral vessels, which are major features of Alzheimer’s disease (AD) and cerebral amyloid angiopathy (CAA), respectively [1, 2]. Both diseases are characterized by devastating clinical presentations together with a high prevalence in the elderly population. In this sense, AD is the first cause of dementia affecting 24 million people worldwide [3, 4]. AD patients show severe β-amyloidosis characterized by the presence of parenchymal Aβ deposits, together with intracellular accumulation of hyperphosphorylated TAU protein (neurofibrillary tangles) triggering neuroinflammation, neurodegeneration, and dementia [2]. CAA, on the other hand, is characterized by Aβ deposition in brain vessels causing dementia and being the leading cause of lobar intracerebral hemorrhage (ICH) in the elderly population [5]. The Aβ peptide is the product of the amyloid precursor protein (APP) processed by β- and γ-secretases, giving rise to peptides of different lengths, including Aβ_40_ and Aβ_42_, which are the most interesting forms from a clinical point of view. Although both Aβ peptides are prone to aggregate-forming amyloid fibrils that deposit in the brain, Aβ_42_ preferentially deposits in the parenchyma (forming neuritic plaques), whereas Aβ_40_ is more commonly associated with CAA-affected vessels [6, 7].

The underlying mechanisms explaining pathological Aβ accumulation in the brain are not completely clear. Defective routes of Aβ elimination have been proposed as an important factor leading to sporadic cerebral β-amyloidosis [8]. In this context, the most relevant Aβ clearance pathways described to date include proteolytic degradation by extracellular proteases, such as insulin-degrading enzyme (IDE) and neprilysin (NEP) [9]; clearance across the blood-brain barrier (BBB) by binding to specific receptors, such as LRP-1 and LRP-2, among others [10]; and through perivascular drainage [11], promoting the mobilization of Aβ material along the basement membrane of cerebral arteries and capillaries to the cervical lymph nodes for its elimination. Finally, Aβ can be eliminated through glial uptake by astrocytes, microglia, and myeloid cells derived from peripheral monocytes followed by intracellular digestion [12–14]. Microglial cells are resident innate immune cells that play an important role in mediating the neuroinflammatory process in the brain and the phagocytosis of toxic species such as Aβ material. In this context, the importance of this dual role of microglia in cerebral β-amyloidosis is increasingly recognized [15]. At this juncture, therapeutic strategies stimulating these elimination pathways have been proposed for AD.

Certain apolipoproteins, beyond their role in lipid and cholesterol metabolism, have been shown to modulate Aβ accumulation in the brain. *APOE* genotype is the strongest genetic risk factor for the development of sporadic cerebral β-amyloidosis [16, 17], and although its interaction with Aβ is not well understood, it has been proposed that apolipoprotein E (ApoE) influences Aβ fibrillization [18], together with its clearance across the BBB [19]. On the other hand, apolipoprotein J (ApoJ, also known as clusterin) is one of the most highly expressed apolipoproteins in the brain after ApoE [20]. A crucial role of ApoJ in cerebral β-amyloidosis is supported by several lines of evidence, although its precise role is not well determined. ApoJ is a heterodimeric multifunctional protein. It has been described as an anti-inflammatory and antiapoptotic protein [21, 22] and a regulator of complement activation [23]. In addition, it is a very effective chaperone that is able to bind to partially misfolded proteins. In this context, ApoJ is able to bind to Aβ and prevent its fibrillization [24] and toxicity in vitro [25]. In AD brains, ApoJ is codeposited with neuritic plaques and CAA-affected vessels, and ApoJ-Aβ complexes have been detected in cerebrospinal fluid (CSF) [26]. The role of ApoJ in AD was further supported by a GWAS (genome-wide association study) that identified the *CLU* locus as a risk factor for developing this neurological disease [27]. More revealingly, the risk factor allele rs11136000^C^ was associated with low expression of ApoJ, whereas the protective allele rs11136000^T^ caused higher ApoJ expression [28]. On the other hand, elevated circulating levels of ApoJ in plasma have been related to the severity and prevalence of AD [29]. This last piece of evidence suggests that an increase in ApoJ levels would occur as a protective response against the aberrant accumulation of Aβ in the brain. However, there are conflicting results in the literature regarding the protective role of ApoJ. This is the case in a study showing that PDAPP mice lacking *Clu* (PDPP/Clu) presented reduced fibrillary Aβ without altering total levels of Aβ [30]. On the other hand, Wojtas and colleagues observed that knocking out *Clu* in APP/PS1 mice resulted in an exacerbation of vascular Aβ deposition, whereas total Aβ and parenchymal Aβ deposition were remarkably reduced [31]. In this sense, more recently, it has been described that the *Clu* deficiency in the 5xFAD mouse AD model reduces the different Aβ pools only in young mice, although this effect disappears with age [32]. Nevertheless, the impact of increasing ApoJ in the circulation and/or in the brain still needs to be clarified.

From this background, our main objective was to understand the role of ApoJ in the development and progression of cerebral β-amyloidosis by determining the potential effect of ApoJ-based therapies in an experimental model of AD. Because ApoJ is present in plasma as free/nonlipidated form or associated with HDL (high-density lipoproteins) particles (20% of circulating ApoJ) [33], we also analyzed whether lipidation of ApoJ could induce functional modifications associated with Aβ distribution in the brain. For this purpose, we analyzed the effect of peripheral administration of human recombinant ApoJ (h-rApoJ) in nonlipidated (free rApoJ) and lipidated (reconstituted HDL nanodiscs formulated with rApoJ, rHDL-rApoJ nanodiscs) [34] structural states, in terms of cerebral Aβ accumulation, neuronal loss, and neuroinflammation in aged APP23 transgenic mice.

## Methods

### Human recombinant ApoJ

The production of functional human recombinant ApoJ (rApoJ) has been previously reported by our group [34, 35]. Briefly, human embryonic kidney 293 T cells (HEK293T) were transfected with the pcDNA4.0™ vector containing the human *APOJ* cDNA (Abgent, Clairemont, San Diego, USA). Stable transfected cells were grown in HYPERFlask systems (Corning Inc., New York, USA) followed by the recollection of cell supernatants for protein purification with Ni-affinity chromatography with fast protein liquid chromatography (FLPC; AKTA Purifier 100 system, GE Healthcare Bio-Sciences Corp., Piscataway, NJ, USA) in HiScreen Ni FF columns (GE Healthcare). The purified protein was dialyzed overnight (ON) at 4 °C against tris buffer saline (TBS) in 10 KDa SnakeSkin Dialysis Tubing membranes (Thermo Fisher, Waltham, MA, USA). We confirmed that the produced human rApoJ was a mature-secreted protein formed as a heterodimeric complex of two 40–45 kDa subunits [34]. For those batches intended to the preparation of reconstituted HDL particles, the resulting purified rApoJ was dialyzed against TBS (pH = 6.4). Finally, the protein concentration was determined by BCA assay (Thermo Fisher) and diluted to a final rApoJ concentration of 200 μg/ml in PBS. Aliquots were stored at − 80 °C until its usage.

### Preparation and purification of rHDL-rApoJ nanodiscs

A detailed protocol for the preparation and purification of reconstituted HDL nanodiscs formulated with rApoJ (rHDL-rApoJ nanodiscs) has been previously published by our group [34]. Briefly, the process started with lipid mixture preparation with 1,2-dimyristoyl-*sn*-glycero-3-phosphocholine (DMPC, Avanti Polar Lipids, Alabaster, AL, USA) and free cholesterol (CHOL, Sigma-Aldrich, Saint Louis, MO, USA) in chloroform solution (5:1 DMPC:CHOL molar ratio). The organic solvent was removed under vacuum and nitrogen to afford a dry lipid film, followed by rehydration with TBS 40 mM sodium deoxycholate (cholate, Sigma-Aldrich). This suspension was incubated 30 min at 37 °C until achieving a clear solution containing the DMPC/CHOL/cholate mixed micelles. For the preparation of rHDL-rApoJ nanodiscs, mixed micelles were incubated with free rApoJ at 550:110:1 DMPC/CHOL/rApoJ molar ratio and three incubation cycles were performed at 4 °C and 37 °C to promote the lipid-protein interaction. After incubation, the rHDL-rApoJ nanodiscs self-assembly started with the cholate removal through extensive dialysis against 1000-fold excess TBS at 4 °C during 48 h in 10 KDa SnakeSkin Dialysis Tubing with two buffer changes (1 × 10^9^ overall dilution factor). Finally, dialyzed samples were centrifuged at 16000×*g* for 30 min at 4 °C to eliminate the unbound lipids. A purification step with potassium bromide (KBr; Sigma Aldrich) density gradient ultracentrifugation was needed to allow the elimination of unbound free rApoJ. This step (100,000×*g*; 24 h, 1250 mg/ml density of KBr), allowed the recollection of purified rHDL-rApoJ in the upper fractions (< 1250 mg/ml density). These samples were dialyzed against PBS-2% sucrose to eliminate KBr. The protein concentration was determined by BCA (Thermo Scientific). rHDL-rApoJ containing solutions were diluted in PBS to a final rApoJ concentration of 200 μg/ml, and aliquots were stored at − 80 °C until its usage.

### Subchronic in vivo administration of rHDL-rApoJ nanodiscs and free rApoJ

Mice were housed in a climate-controlled environment on a 12/12-h light/dark cycle with food and water available ad libitum. The effect of the subchronic administration of both rHDL-rApoJ nanodiscs and free rApoJ was studied in male APP23 transgenic mice (Hemizygote B6, D2-TgN[Thy-APPSWE]-23-Tg mice, Novartis, Basel, Switzerland) [36]. Male APP23 mice were used in order to diminish variation in age-related Aβ deposit quantification, since male and female APP23 mice differ in the age at which Aβ deposition begins. These transgenic mice overexpress the APP protein with the Swedish mutation (K670M/N671L) under the murine neuronal *Thy1* promoter (thymocyte antigen-1). Hemizygous APP23 mice were backcrossed with C57BL/6 mice (Janvier Labs, Le Genest-Saint-Isle, France), and the APP genotype was tested in the offspring by Transnetyx (Cordova, TN, USA). Wild-type (wt) and APP23 mice were aged in the animal facility of our institution to obtain the final study cohort. APP23 mice received 8 intravenous (IV) doses of rHDL-rApoJ or free rApoJ (1 mg/kg of rApoJ) or saline for 4 weeks (2 doses/week). The age (mean ± SD) at the beginning of the treatment for each APP23 group was 14.00 ± 0.73 months old for the saline-treated mice (*N* = 7), 14.07 ± 0.75 months old for the rHDL-rApoJ-treated mice (*N* = 7) and 13.99 ± 0.47 months old for the free rApoJ-treated mice (*N* = 7). For the administration of treatments, mice were anesthetized with isoflurane (5% for induction and 2% for maintenance in medicinal air/oxygen, Abbot Laboratories, Spain) followed by retro-orbital sinus injection, selected as a reliable administration route for IV delivery [37]. All animals completed the treatment, which was not associated with mortality or body weight loss. All assays conducted in mouse brains after treatment were evaluated in a blinded manner.

### Cerebrospinal fluid collection

Thirty minutes after the last administration, mice were anesthetized under isoflurane and CSF was sampled as previously described [38]. Briefly, anesthetized mice were placed in the stereotaxic instrument and a sagittal section inferior of the occiput was done in the skin. Once the dura mater of the cistern magna was visualized under the microscope, it was penetrated with a glass capillary (inner diameter of 0.5 mm) and clear CSF ascended by capillarity. Only completely clear CSF was considered for further experiments.

### Blood and brain collection

Blood was collected through cardiac puncture, and blood samples were centrifuged in ethylenediaminetetracetic acid (EDTA) tubes to recollect the EDTA-plasma. Then, after transcardial perfusion with 25 ml of cold PBS (pH = 7.4), brains were rapidly removed and divided into two hemispheres. One of the hemispheres was frozen at − 80 °C for homogenization, while the other was fixed in 10% formalin (Diapath, Martinengo, Italy) at room temperature (RT) for 48 h before paraffin embedding.

### Brain homogenates

One of the hemispheres of each mouse was homogenized to obtain soluble, membrane-associated, and insoluble fractions through sequential centrifugations. First, the brain tissue was homogenized in 4 ml of TBS (pH = 7.4) supplemented with cOmplete EDTA-free protease inhibitor cocktail (Roche Diagnostic, Mannheim, Germany) and samples were centrifuged at 8000×*g* for 30 min a 4 °C. The supernatant was selected as the soluble fraction whereas the pellet was homogenized in TBS supplemented with TritonX-100 and protease inhibitors followed by centrifugation at 8000×*g* for 30 min at 4 °C. The supernatant was selected as the membrane-bound fraction whereas the pellet was homogenized in 5 M guanidine-HCl (pH = 8, Sigma-Aldrich) supplemented with protease inhibitors. Samples were shaken for 3 h at RT followed by centrifugation at 8000×*g* for 30 min at 4 °C, and the supernatant was selected as the insoluble fraction. Soluble, membrane-bound, and insoluble fractions were aliquoted and frozen at − 80 °C. The protein concentration of each fraction was quantified by BCA assay (Thermo Fisher).

### Enzyme-linked immunosorbent assay (ELISA)

Aβ levels in plasma, CSF, and soluble and insoluble brain fractions were quantified with commercial ELISA kits for both Aβ_40_ (KHB3481) and Aβ_42_ (KHB2442, Thermo Fisher). Levels of human ApoJ in plasma of treated mice were quantified with the commercial ELISA kit (3713-1HP, Mabtech, Nacka Strand, Sweden). Levels of the endogenous mouse ApoJ in brain homogenates and plasma in APP23 mice were determined using the mouse Clusterin ELISA Kit (ab199079, Abcam, Cambridge, UK). Commercially available multiplexed ELISA was used to investigate the general inflammatory state in brain soluble fraction after treatments (Mouse cytokine magnetic 20-plex panel, LMC0006M, Invitrogen). Results were analyzed in duplicate with Luminex technology in a MAGPIX™ instrument, and samples with a coefficient of variation (CV) > 35% were excluded. In all cases, data were corrected by the amount of total protein determined by BCA.

### Immunohistochemistry

A series of three paraffin-embedded sagittal sections were selected 800 μm apart, starting from the division between the hemispheres. For the detection of Aβ with conventional immunohistochemistry (IHC), all samples were deparaffinized for 1 h at 65 °C, rehydrated, and treated with 2% H_2_O_2_ and 10% methanol in PBS for 15 min. The samples were incubated for 1 h in blocking solution (PBS, 0.2% Triton-X, 10% fetal bovine serum (FBS, Millipore, Darmstadt, Germany) followed by ON incubation with the anti-Aβ monoclonal antibody 4G8 (BioLegend, San Diego, CA, USA) diluted 1:10000 in blocking solution. Next, slices were incubated with biotinylated anti-mouse IgG (Vector Laboratories, Burlingame, CA, USA) diluted 1:200 in blocking solution for 1 h at RT and with streptavidin-HRP (Vector Laboratories) diluted 1:200 in blocking solution for 1 h at RT. Finally, diaminobenzidine (DAB; Dako, Denmark) was applied to the samples until a brown end-product was observed. Contrast staining was performed by immersion of the samples in Harris hematoxylin solution (Sigma-Aldrich). To quantify the number of Aβ-positive deposits, we used the overview tool of a Leica LMD 6500 Laser Microdissection Microscope System to scan each brain slice in full. Overview images were labeled with codes to ensure blind quantification. The number of Aβ deposits larger than 50 px^2^ in the cortex and the area (px^2^) occupied per deposit were quantified with ImageJ software. Three different deep sections per brain were analyzed, and a mean of the values determined in those sections was obtained. Data are expressed as the number of Aβ-positive deposits per total area (px^2^) and the mean size of those Aβ-positive deposits. Representative images were obtained with a conventional microscope (BX61, Olympus, Tokyo, Japan).

An immunohistochemistry protocol was also performed for the quantification of NeuN-positive cells in the cortex and hippocampus (CA1, CA2/CA3, and polymorphic layer of dentate gyrus (poDG)) of APP23 and age-matched wt mice in the first deep-section slice. Before the blocking step, an antigen retrieval step was performed by immersing the slices in citrate buffer (10 mM sodium citrate, 0.05% TWEEN-20, pH = 6) at 95 °C for 30 min. The NeuN antibody (Millipore) was diluted 1:200 in blocking solution. The quantification of NeuN-positive cells in the different areas was performed as previously described [39]. Briefly, for each sample and brain region, three different images were taken at × 40 magnification using an LMD6500 microscope (Leica, Wetzlar, Germany). NeuN-positive cells were counted in three different squares in every image measuring 150 × 150 μm in cortical regions, whereas squares measuring 100 × 100 μm were used in hippocampal regions. The results are expressed as the number of NeuN-positive cells per square millimeter.

### Thioflavin S staining

Thioflavin S (ThS) staining was performed to detect fibrillar Aβ deposits in brain slices. After deparaffination and rehydration, a series of 3 selected sagittal sections 800 μm apart were immersed in ThS (Sigma-Aldrich) 1% solution in 75% ethanol for 30 s. The excess of ThS was drained, and the sections were immersed in ThS 0.1% in 75% ethanol for 1 min. Finally, the slices were dehydrated, and DAPI (4′,6-diamidino-2-phenylindole) was used for counterstaining before the preparations were mounted. The number of ThS-positive deposits, the size of those deposits, and the number of ThS-positive brain vessels were quantified as previously described for the quantification of Aβ deposits by immunohistochemistry. The count of ThS-positive deposits larger than 50 px^2^ and the analysis of the area (px^2^) occupied per deposit were quantified with ImageJ software. Data are expressed as the number of CAA-affected vessels (ThS-positive brain vessels), the number of ThS-positive deposits per total area (px^2^) and the mean size of ThS-positive deposits. Representative images were obtained with a conventional fluorescence microscope (BX61, Olympus, Tokyo, Japan).

### Immunofluorescence

A cluster of differentiation 68 (CD68) as a marker of phagocytosis and Tomato lectin (TL) as vascular and microglia marker [40] were analyzed through immunofluorescence in paraffin-embedded brain sections. To this end, after deparaffination, rehydration, and antigen retrieval with citrate buffer (10 mM sodium citrate, 0.05% Tween20, pH = 6, 30 min at 95 °C), slices were blocked in PBS pH = 7.4 supplemented with 10% FBS and 1% Tween 20 for 1 h at RT. Rabbit anti-mouse CD68 antibody (Abcam) diluted 1:50 in blocking solution was incubated ON at 4 °C. Next, secondary antibody anti-rabbit-AlexaFluor488 (Thermo Fisher) diluted 1:500 in blocking solution was incubated 1 h at RT followed by incubation with Tomato lectin-DyLight564 (Vector Laboratories) diluted 1:100 in blocking solution 1 h at RT. On the other hand, the localization of CD68 and Aβ was analyzed in paraffin-embedded brain sections. After the deparaffination and rehydration steps, brain slices were treated with 70% formic acid for 10 min followed by 3 washes of 5 min with PBS-1% Tween20. Then, the samples were incubated in blocking solution for 1 h at RT followed by ON incubation at 4 °C with mouse anti-human Aβ monoclonal antibody (4G8) diluted 1:1000 and rabbit anti-mouse CD68 (Abcam) diluted 1:50 or rabbit anti-Iba1 (ionized calcium binding adapter molecule-1, Abcam) diluted 1:100 in blocking solution. Next, secondary antibodies anti-rabbit-AlexaFluor-488 and anti-mouse-AlexaFluor694 (Thermo Fisher) diluted 1:500 in blocking solution for 1 h at RT. Finally, contrast staining was done with DAPI before mounting the preparations. Images were obtained with a conventional fluorescence microscope (BX61, Olympus). Finally, human ApoJ was analyzed in brain sections of treated animals. To this end, after the rehydration, antigen retrieval, and blocking steps, brain slices were incubated ON with mouse-anti-human ApoJ (BD Bioscience, San Jose, CA, USA) diluted 1:100 in blocking solution. AlexaFluor-594 was used as a secondary antibody (1:500), and DAPI was used as contrast staining. Images were obtained with a confocal spectral microscopy (FV1000, Olympus).

### Western blot

For the immunodetection of different protein profiles, 40 μg of soluble or membrane-bound brain homogenate fractions or 1 μl of mouse plasma was loaded in 10% acrylamide SDS-PAGE under reducing conditions (5% β-mercaptoethanol and sample heating at 95 °C). Then, gels were transferred onto nitrocellulose membranes using Trans-Blot Turbo transfer system (Bio-Rad, Hercules, CA, USA) at 1.3 A, 25 V for 10 min and blocking was done in 10% non-fatted milk for 1 h at RT. Membranes were incubated in tested specific antibodies diluted in blocking solution at 4 °C ON. Suitable secondary HRP-labeled antibodies diluted in blocking solution were incubated 1 h at RT. The processing of membranes continued with the incubation with PierceECL Western blotting Luminol/Enhancer and Stable Peroxidase solutions (Thermo Fisher). The relative amount of the targeted protein was calculated from the quantification of the mean intensity with the ImageJ software. Immunoblotting of β-actin, β-tubulin, or glyceraldehydes 3-phosphate dehydrogenase (GADPH) was used as loading controls. The primary antibodies used were rabbit anti-CD68 (1:1000, Abcam), rabbit anti-GFAP (Glial Fibrillary Acidic Protein, 1:1000, Dako), rabbit anti-Iba1 (1:1000, Abcam), mouse anti-LRP1 (1:1000, Abcam), mouse anti-human APP (1:1000, Millipore), mouse anti-sAPPα (Soluble APP fraction-α, 1:1000, Clontech, Mountain View, CA, USA), mouse anti-sAPPβ (Soluble APP fraction-β, 1:1000, Clontech), rabbit-anti RAGE (1:1000, Abcam), goat-anti-mouse ApoJ (1:1000, RD Systems, Minneapolis, MN, USA), mouse anti-β-actin (1:10000, Sigma-Aldrich) and mouse anti-GADPH (1:10000, Invitrogen). The following HRP-labeled secondary antibodies were used: Anti-rabbit-HRP (1:2000, GE Healthcare Biosciences, Little Chalfont, UK), anti-mouse-HRP (1:2000, GE Healthcare Bioscience), and anti-goat-HRP (1:5000, Sigma-Aldrich).

### Microglial cell cultures

The mouse microglial cell line BV-2 was obtained from the European Collection of Authenticated Cell Culture (ECACC). BV-2 cells were seeded (30.000 cells/well) in poly-l-lysine (Sigma-Aldrich)-coated sterile coverslips in 24-well plates and incubated at 37 °C in RPMI 1640 medium (Thermo Fisher) supplemented with 10% fetal bovine serum (FBS, Thermo Fisher) and 1% penicillin-streptomycin (Thermo Fisher) for 24 h. For treatments, the medium was changed to RPMI containing 0.1% FBS and cells were incubated with or without rApoJ (0.1 μM) for 24 h as a pre-treatment. Then, rApoJ was removed by washing the wells with RPMI 0.1% FBS and 0.5 μM human Aβ(1–40) HiLyteTM Fluor 488-labeled (AnaSpec, Fremont, CA, USA) was added for 3 h to the corresponding wells. Next, cells were washed twice with PBS containing 5% bovine serum albumin (BSA, Sigma-Aldrich) and fixed with 4% paraformaldehyde (PFA, Sigma-Aldrich) for 15 min. After fixation, cells were washed twice with PBS and levels of 488-Aβ(1–40) in the cells were analyzed by measuring the fluorescence (Ex/Em = 503/528 nm) using a Synergy Mx microplate reader (Biotek). To visualize the cellular structure, a phalloidin-tetramethylrhodamine B isothiocyanate (phalloidin-TRITC, Sigma-Aldrich) actin staining was used (50 μg/ml in PBS for 20 min). After washing twice with PBS, the coverslips were mounted on slides using FluoroshieldTM with DAPI (Sigma-Aldrich). Images were obtained with confocal spectral microscopy (FV1000, Olympus).

### Statistical analysis

GraphPad Prism 6 and SPSS Statistics 17.0 software were used for the statistical analysis. The normality of distributions was assessed with the Shapiro-Wilk normality test. The differences were assessed using a *t* test or one-way ANOVA with an LSD (least significant difference) post hoc test when appropriate. When data did not fit a normal distribution, the Kruskal–Wallis test with a Dunn’s post hoc was used for multiple comparisons, and the Mann–Whitney test was used for one-to-one comparisons. The Benjamini-Hochberg false discovery rate (FDR) correction was used to control for multiple testing in the multiplexed analysis of brain inflammatory status. A *p* value < 0.05 was considered statistically significant.

## Results

**Fig. 1:**
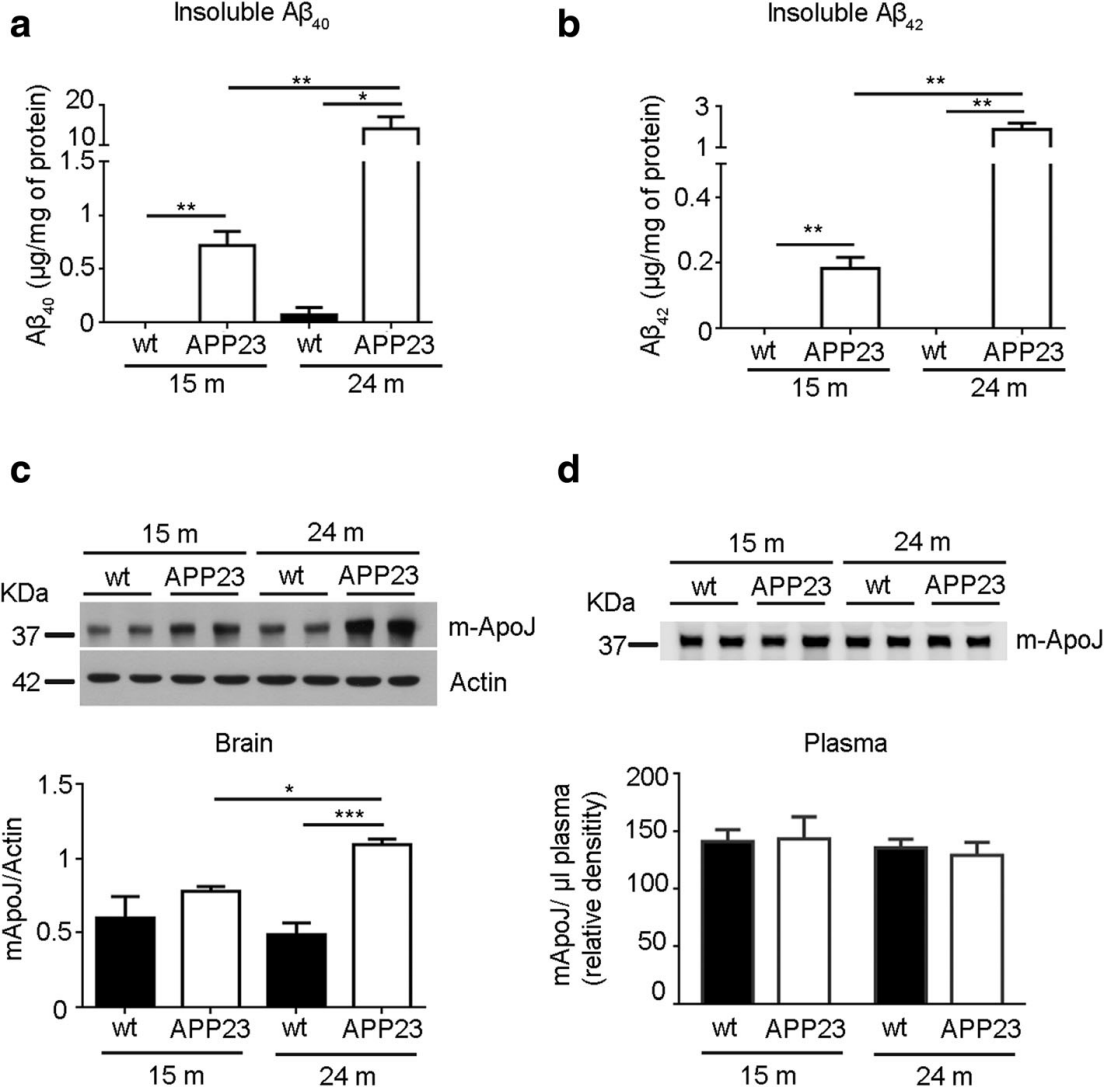
Levels of cerebral insoluble Aβ and mouse ApoJ expression in APP23 transgenic mice over time. **a** Cerebral accumulation of insoluble Aβ_40_ in 15- and 24-month-old APP23 and wt mice. **b** Cerebral accumulation of insoluble Aβ_42_ in 15- and 24-month-old APP23 and wt mice. **c** Mouse ApoJ (m-ApoJ) cerebral expression in 15- and 24-month-old APP23 and wt mice. **d** Mouse ApoJ (m-ApoJ) levels in the plasma of 15 and 24-month-old APP23 mice. Data are expressed as the mean ± SEM. *N* = 3–4/group. *p < 0.05; **p < 0.01; *p < 0.001

APP23 mice are characterized by increasing accumulation of both human Aβ_40_ and Aβ_42_ in the brain. This rising Aβ deposition became especially evident in very old APP23 mice (24 months old), where a 20-fold increase in insoluble Aβ_40_ and a 10-fold increase in insoluble Aβ_42_ were observed in comparison with the corresponding Aβ load in 15-month-old APP23 mice (Fig. 1a, b). Interestingly, this growing Aβ accumulation was accompanied by an increase in mouse ApoJ (m-ApoJ) levels in the brain (Fig. 1c). Twenty-four-month-old APP23 mice presented significantly higher m-ApoJ brain levels than age-matched wt mice, whereas the difference between genotypes was nonsignificant in 15-month-old mice. In addition, as expected, this increase in brain m-ApoJ was exacerbated with age, as significant differences in m-ApoJ levels were detected between 15- and 24-month-old APP23 mice. Plasma levels of endogenous m-ApoJ were also determined, although no significant differences between genotypes were found in 15- or 24-month-old mice (Fig. 1d).

**Fig. 2:**
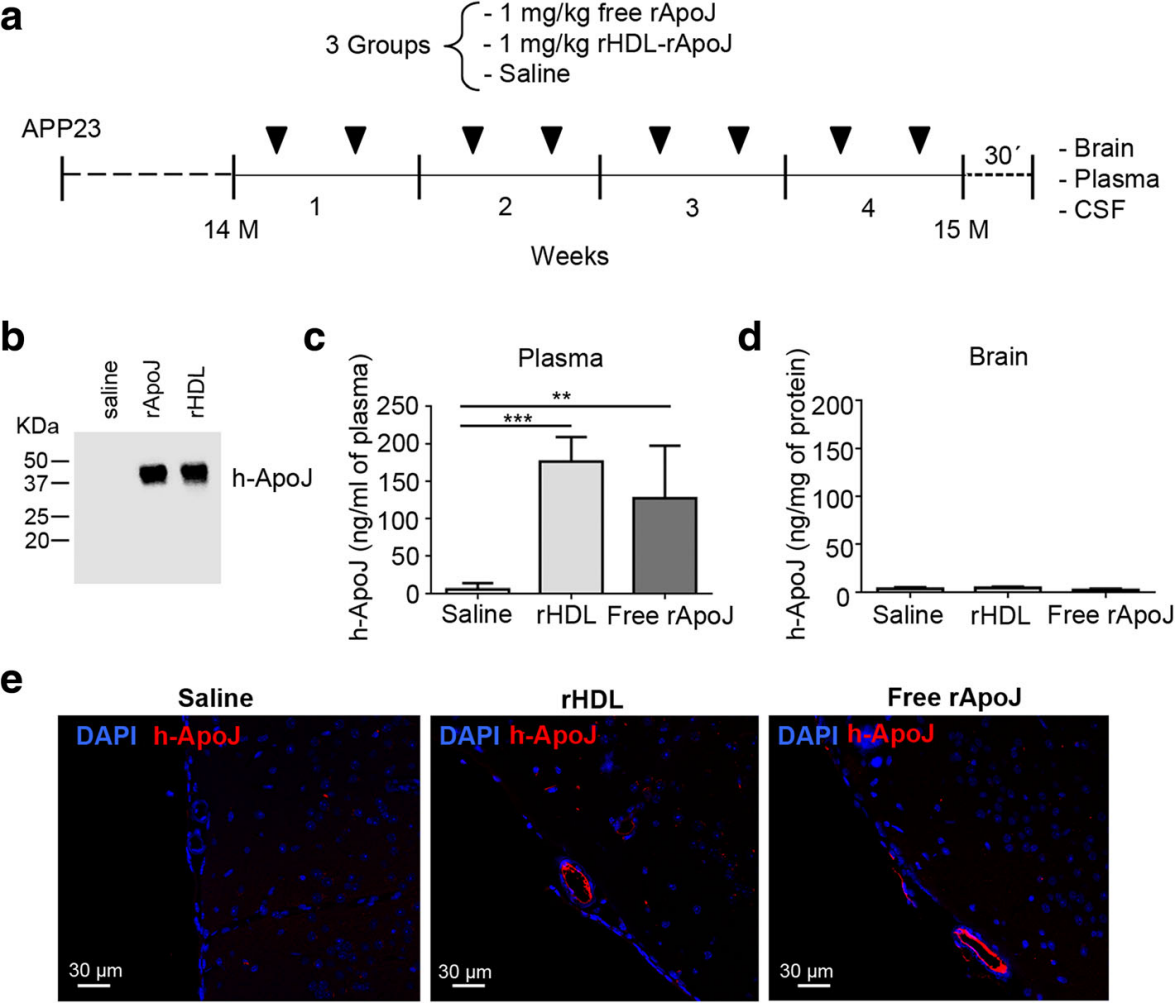
Subchronic intravenous treatment of APP23 mice with human recombinant ApoJ (h-rApoJ). **a** Schematic timeline representing the experimental design of the study based on the subchronic administration of APP23 mice with rHDL-rApoJ nanodiscs, free rApoJ or saline. **b** Immunodetection (anti-h-ApoJ) of treatment samples before being intravenously infused. **c** Plasmatic levels of h-ApoJ in treated mice 30 min after the last administration detected by ELISA (*N* = 7/group). **d** Levels of h-ApoJ in brain homogenates from treated mice detected by ELISA (*N* = 7/group). **e** Immunofluorescent detection of h-ApoJ in paraffin-embedded brain sections. **p < 0.01; ***p < 0.001

The endogenous increase in m-ApoJ in APP23 mice at late pathological stages could be interpreted as a physiological protective mechanism to promote reduction and clearance of Aβ in the brain. In this regard, we wondered whether an earlier increase in ApoJ levels could be a beneficial strategy to delay the Aβ deposition detected over time. Therefore, our main objective was to study the effect of peripheral administration of free rApoJ or lipidated rApoJ (rHDL-rApoJ nanodiscs) in APP23 mice. To this end, APP23 mice received 8 intravenous administrations (over the course of 1 month) of free rApoJ (1 mg/kg), rHDL-rApoJ (1 mg/kg), or saline, with 2 infusions/week (Fig. 2a). The administered dose of 1 mg/kg of human rApoJ would theoretically lead to a mean increase of 20% in total circulating ApoJ levels considering the m-ApoJ levels determined in plasma. The equality of the initial amounts of infused rHDL-rApoJ and free rApoJ was confirmed by Western blot, as shown in Fig. 2b, where the α- and β-chains of rApoJ were observed at ≈ 37 KDa. Next, the circulating concentration of human rApoJ (h-rApoJ) was quantified by ELISA. A significant increase in plasma levels of h-rApoJ was observed in both rHDL-rApoJ- and free rApoJ-treated mice, whereas human ApoJ, as expected, was not detected in the saline group (Fig. 2c). In addition, no significant differences in h-ApoJ between the rHDL-rApoJ- and free rApoJ-treated groups were found in plasma 30 min after administration. On the other hand, the levels of h-rApoJ in brain homogenates were undetectable by ELISA (< 70 pg/ml) 30 min after the last administration of the subchronic treatment (Fig. 2d). However, immunofluorescence analysis revealed specific staining for h-ApoJ in occasional vessels from the brains of both rHDL-rApoJ- and free rApoJ-treated mice. Brains from saline-treated mice did not present human ApoJ staining (Fig. 2e). On the other hand, h-rApoJ-based peripheral treatments did not influence the expression of m-ApoJ in brain homogenates or m-ApoJ levels in plasma, as detected by ELISA (Additional file 1: Figure S1).

**Fig. 3:**
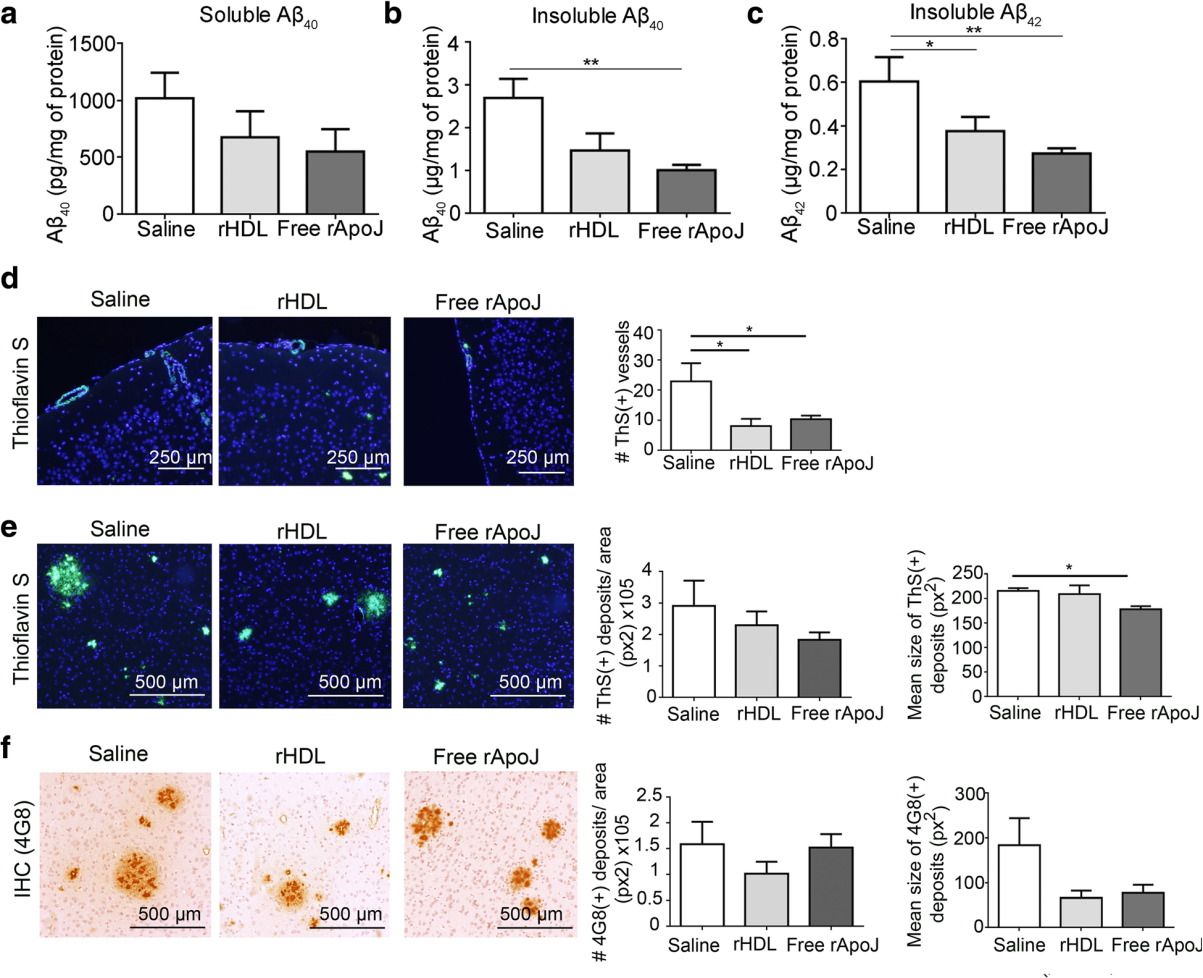
Cerebral Aβ levels in 15-month-old APP23 mice treated subchronically with saline, rHDL-rApoJ nanodiscs, or free rApoJ. **a** Cerebral soluble Aβ_40_ levels. **b** Cerebral insoluble Aβ_40_. **c** Cerebral insoluble Aβ_42_, detected by ELISA. **d** Representative images showing ThS-positive vessels and the corresponding quantification in brain sections. **e** Representative images showing ThS-positive deposits, quantification of the number of ThS-positive deposits per area (px^2^), and quantification of the mean size of those deposits. **f** Representative images of anti-Aβ (4G8)-positive deposits in the brain parenchyma, quantification of the number of Aβ-positive deposits per area (px^2^), and quantification of the mean size of those deposits. Data are expressed as the mean ± SEM. *N* = 7/group. *p < 0.05; **p < 0.01

The efficacy of subchronic rApoJ-based treatments in APP23 mice was first evaluated in terms of cerebral Aβ load. Levels of Aβ_40_ and Aβ_42_ in soluble and insoluble fractions of brain homogenates were quantified by ELISA after the treatments (Fig. 3a–c). The levels of soluble Aβ_40_ were not significantly different among treatments, although a trend towards a reduction in free rApoJ-treated mice was observed (*p* = 0.09). Soluble Aβ_42_ levels were under the lower limit of detection (< 6 pg/ml). Interestingly, we observed lower levels of both insoluble Aβ_40_ and Aβ_42_ in mice treated with free rApoJ than in the saline-treated group (insoluble Aβ_40_ 1.01 ± 0.12 vs. 2.70 ± 0.44 μg/mg and insoluble Aβ_42_ 0.27 ± 0.02 vs. 0.60 ± 0.11 μg/mg in rApoJ-treated group compared to the saline-treated group, *p* < 0.01) (Fig. 3b, c). However, after treatment with rHDL-rApoJ nanodiscs, the insoluble Aβ_40_ levels remained unaltered, whereas lower insoluble Aβ_42_ brain levels were found in comparison with the saline-treated group (insoluble Aβ_42_ 0.38 ± 0.07 vs. 0.60 ± 0.11 μg/mg in rHDL-treated group compared to the saline-treated group, *p* < 0.05) (Fig. 3b, c). Regarding the cerebral Aβ distribution, the number of Aβ-affected vessels and the number of parenchymal deposits were quantified by ThS staining and immunohistochemistry with a specific anti-Aβ antibody (Fig. 3d–f). Brains from mice treated with rHDL-rApoJ and free rApoJ presented a significantly lower number of CAA-affected arteries as detected by ThS staining (the reduction percentages were 64.9% and 54.8% vs. saline-treated group, respectively) (Fig. 3d). Although no differences were detected in the parenchymal Aβ deposition load after rApoJ-based treatments (Fig. 3e, f), the mean size of fibrillar Aβ accumulations (ThS-positive deposits) was reduced in those mice treated with free rApoJ (the reduction percentage in free rApoJ vs. saline-treated group was 17.5%) (Fig. 3e). Finally, the deposition load and size of parenchymal Aβ deposits did not differ significantly among groups as shown by a specific immunostaining against Aβ, although a clear tendency towards smaller deposits after both rApoJ-based treatments was again observed (Fig. 3f).

**Fig. 4:**
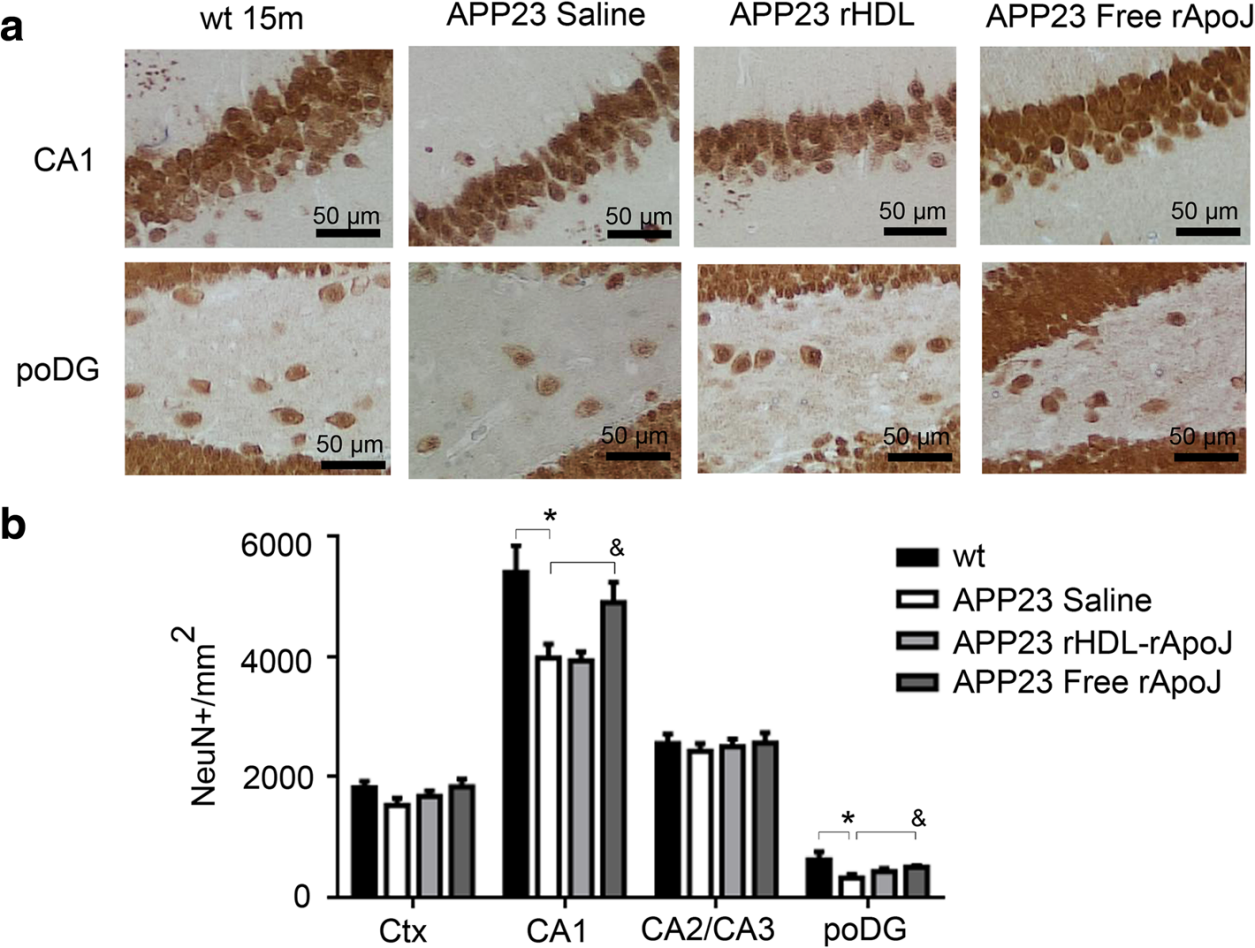
Neuronal loss in 15-month-old wt mice and APP23 mice treated subchronically with saline, rHDL-rApoJ nanodiscs or free rApoJ. **a** Representative images of NeuN-positive cells in CA1 region of the hippocampus and in poDG of APP23-treated mice and wt mice. **b** Quantification of NeuN-positive cells per square millimeter in the different studied regions (cortex, CA1, CA2/CA3, and poDG). Abbreviations: Ctx: Cortex; poDG: polymorphic layers of the dentate gyrus. Data are expressed as the mean ± SEM. *N* = 6–7/group. *p < 0.05 (comparison between WT and saline-APP23 groups) and *p* < 0.05 (comparison among APP23-treated groups)

Neuronal loss is one of the neuropathological hallmarks in AD [41], and it has been described that the APP23 mice model shows a neuronal loss in the CA1 region of the hippocampus and in the poDG from 12 months of age [39]. Thus, we measured the effect of rHDL-rApoJ and free rApoJ treatments over the neurodegeneration in the cortex and hippocampus through NeuN immunostaining. In comparison with age-matched wt mice, we found that 15-month-old APP23 mice exhibited a neuronal reduction in CA1 and poDG (Fig. 4). Importantly, the treatment with free rApoJ was able to significantly ameliorate the neuronal loss in both CA1 and poDG in comparison to the saline-treated group (Fig. 4).

**Fig. 5:**
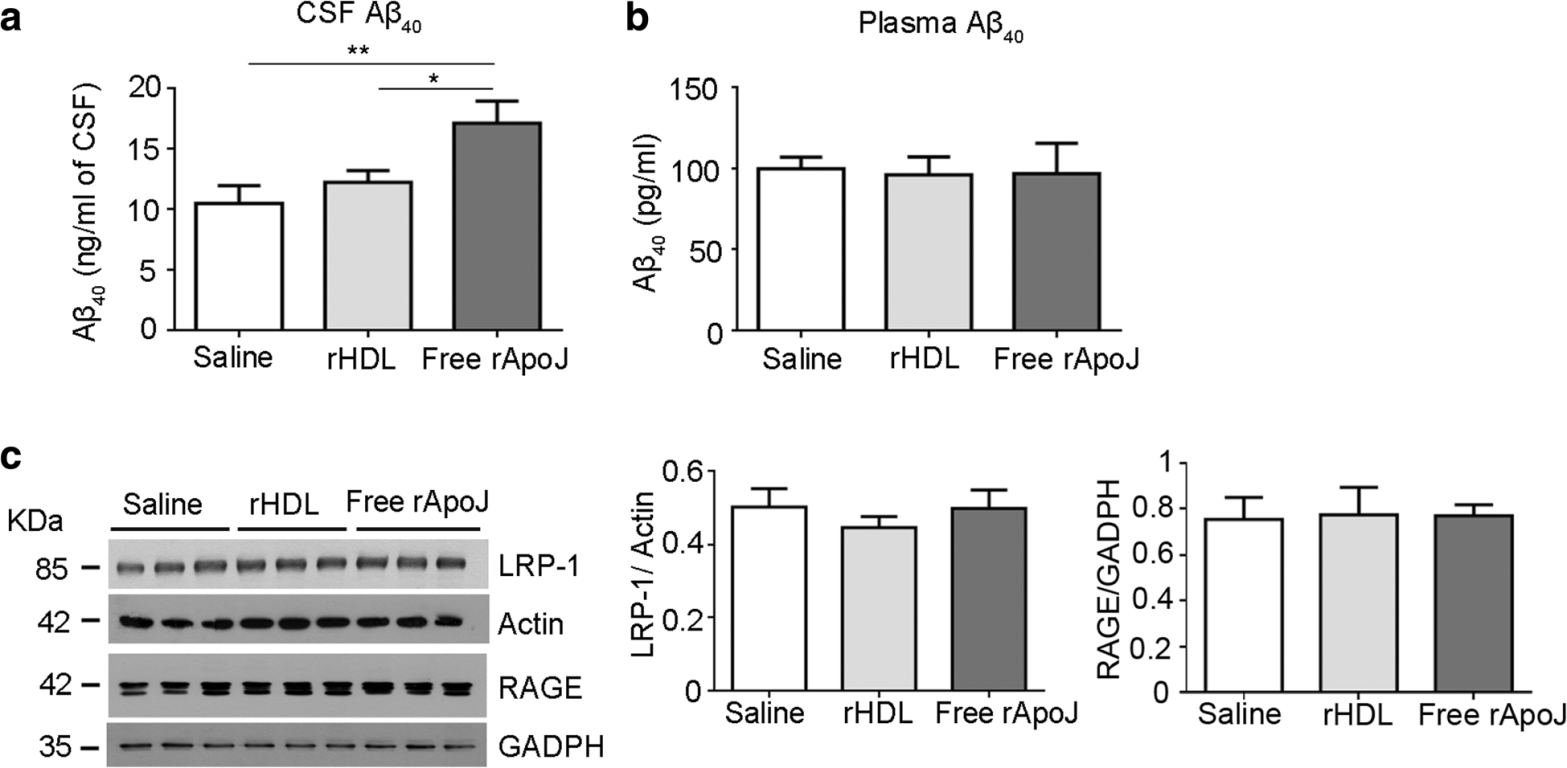
Effect of rHDL-rApoJ nanodiscs or free rApoJ treatment on circulating Aβ levels. **a** CSF levels of Aβ_40_ detected by ELISA. **b** Plasmatic Aβ_40_ levels detected by ELISA. **c** Expression of Aβ-transporting receptors (LRP-1 and RAGE) determined by Western blot. Data are expressed as the mean ± SEM. *N* = 5–7 group. *p < 0.05; **p < 0.01

Because an increase in fibrillary Aβ in the brain is associated with low levels of particular Aβ species in the CSF of AD and CAA patients (Aβ_42_ and Aβ_40_, respectively [42, 43]), we analyzed the Aβ levels in CSF 30 min after the last treatment in APP23 mice. We observed a significant increase in CSF Aβ_40_ levels in mice treated with free rApoJ compared to the rest of the groups (17.10 ± 1.83 vs. 10.46 ± 1.47 ng/ml in the saline-treated group, *p* < 0.01) (Fig. 5a). However, Aβ_40_ levels in plasma were not different among groups 30 min after the last infusion (Fig. 5b). Circulating levels of Aβ_42_ in APP23 mice were undetectable in all cases. In addition, we explored the expression of different brain receptors previously described to mediate the transport of Aβ across the BBB [10]. In this context, the expression of LRP-1 or RAGE did not exhibit expression differences among groups (Fig. 5c). Moreover, we also eliminated the possibility that the decrease in insoluble Aβ in the brain and the increase in CSF Aβ levels obtained after peripheral rApoJ treatment could be explained by an alteration of APP transgene expression or the Aβ peptide production process. Specifically, we confirmed that the expression of APP was unaltered in the brains of mice receiving different treatments, and the processing of APP mediated by α-secretase, β-secretase, and γ-secretase was also unchanged among groups (Additional file 1: Figure S2).

**Fig. 6:**
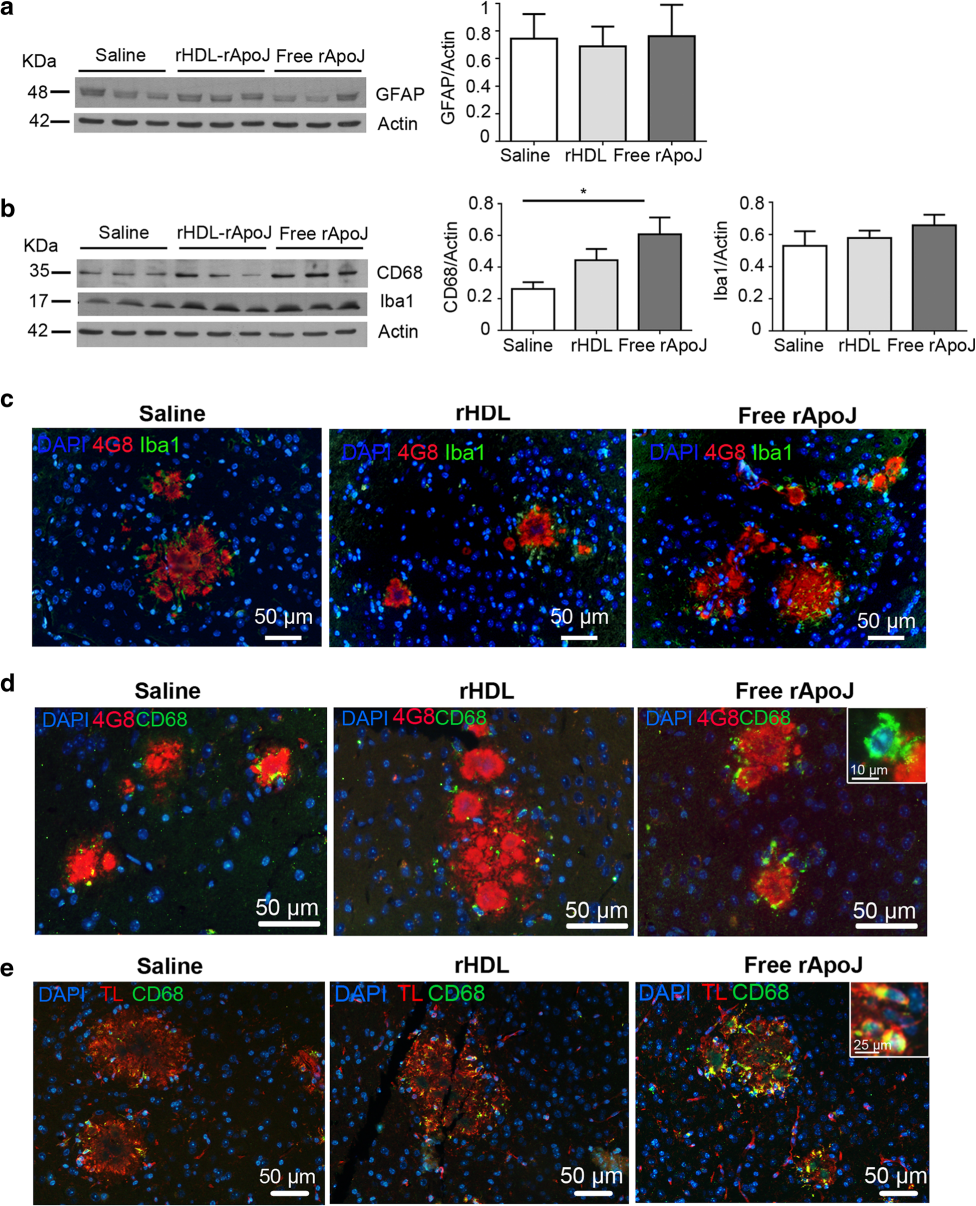
Levels of astrocytic and microglial markers in APP23 mice treated subchronically with saline, rHDL-rApoJ nanodiscs, or free rApoJ. **a** Expression of the reactive astrocyte marker GFAP in mouse APP23-brain homogenates after treatments. **b** Expression of microglial markers (CD68 and Iba-1) in mouse APP23-brain homogenates after treatments. Data are expressed as the mean ± SEM. *N* = 6–7/group. *p < 0.05. **c** Representative immunofluorescence images showing the localization of Iba1 and **d** representative immunofluorescence images showing the localization of CD68 around anti-Aβ (4G8)-positive deposits in brain slices from APP23 mice. **e** Representative immunofluorescence images showing the localization of CD68 with tomato lectin (TL, as a microglial marker) in brain slices from APP23 mice

The neuroinflammatory state of mice that received ApoJ-based treatments was first analyzed by exploring the expression levels of specific cellular markers in brain homogenates. On this front, we determined that the presence of the activated astrocytic protein GFAP did not differ after treatments (Fig. 6a). On the other hand, we detected a 2.3-fold increase in the levels of the lysosomal marker CD68 in brain homogenates from free ApoJ-treated mice (*p* < 0.05), whereas the expression of the resident microglial marker Iba-1 was not significantly different among groups (Fig. 6b). Iba1 and CD68 localization was determined by immunofluorescence, and the signal was observed in the periphery of parenchymal Aβ-positive deposits, showing elevated CD68 expression levels in mice treated with free rApoJ (Fig. 6c, d). Furthermore, double staining of CD68 with tomato lectin (TL) confirmed the presence of this lysosomal marker in microglial cells (Fig. 6e). Additionally, a multiplexed ELISA was performed to detect the global inflammatory state in the brain parenchyma (the complete list of molecules analyzed can be found in Additional file 1: Table S1). Despite the phagocytic phenotype shown by microglial cells after the free rApoJ treatment, we did not observe an increase in the levels of prevalent brain cytokines and inflammatory molecules, such as the classical mediator interleukin 1-β (IL-1β) or tumor necrosis factor-α (TNF-α). In fact, we detected reduced levels of interleukin 17 (IL17) and KC (keratinocyte chemoattractant chemokine) in the brains from mice treated with both rHDL-rApoJ and free rApoJ. However, after FDR correction, only levels of IL17 and KC in free r-ApoJ-treated brains remained statistically different than the corresponding levels in the saline-treated group (Table 1).

**Table 1:** Effect of rHDL-rApoJ nanodiscs or free rApoJ on brain inflammatory status, analyzed by multiplexed ELISA. Data are expressed as the mean ± SEM. *N* = 5–7/group. *p < 0.05 and **p < 0.01 refer to statistical differences vs. the saline-treated group determined by one-way ANOVA test (plus multiple comparison test). The false discovery rate (FDR) correction was used to control for multiple testing in the multiplexed analysis of brain inflammatory status

Inflammatory markers	APP23 saline (pg/mg)	APP23 rHDL-rApoJ (pg/mg)	APP23 free rApoJ (pg/mg)	One-way ANOVA (3 groups) *p* value	rHDL-rApoJ vs saline FDR-corrected *p* value	Free rApoJ vs saline FDR-corrected *p* value
FGF-basic	984.4 ± 154.6	895.6 ± 113.3	560.2 ± 151.4	*p* = 0.108	*p* = 0.787	*p* = 0.174
IL-1β	85.28 ± 12.17	50.86 ± 3.75	72.91 ± 11.43	*p* = 0.080	*p* = 0.122	*p* = 0.448
IL2	151.0 ± 13.09	116.7 ± 14.22	125.2 ± 14.49	*p* = 0.222	*p* = 0.198	*p* = 0.324
IL5	141.7 ± 26.35	130.61 ± 27.54	148.0 ± 21.31	*p* = 0.893	*p* = 0.819	*p* = 0.874
IL6	203.1 ± 22.15	177.7 ± 28.82	176.3 ± 19.5	*p* = 0.656	*p* = 0.583	*p* = 0.448
IL13	21.31 ± 3.40	17.68 ± 2.34	12.63 ± 3.22	*p* = 0.171	*p* = 0.552	*p* = 0.183
IL17	20.01 ± 2.33	13.9 ± 1.37*	10.23 ± 1.08**	*p = 0.004*	*p* = 0.122	*p = 0.017*
IFN-γ	127.8 ± 15.53	89.81 ± 13.28	94.08 ± 10.68	*p* = 0.123	*p* = 0.137	*p* = 0.194
IP10	54.4 ± 4.34	44.48 ± 5.11	46.71 ± 5.05	*p* = 0.330	*p* = 0.289	*p* = 0.388
KC	1558 ± 115.5	1206 ± 112.0*	1043 ± 104.6**	*p = 0.012*	*p* = 0.122	*p = 0.029*
MIG	81.65 ± 8.21	57.81 ± 6.91	61.48 ± 8.72	*p* = 0.090	*p* = 0.122	*p* = 0.194
TNF-α	127.2 ± 13.46	125.2 ± 9.43	144.7 ± 17.79	*p* = 0.620	*p* = 0.937	*p* = 0.448
VEGF	17.67 ± 2.36	14.27 ± 2.35	10.72 ± 1.22	*p* = 0.080	*p* = 0.399	*p* = 0.129
MCP-1	36.1 ± 3.93	24.31 ± 2.84	28.19 ± 3.12	*p* = 0.060	*p* = 0.122	*p* = 0.194

**Fig. 7:**
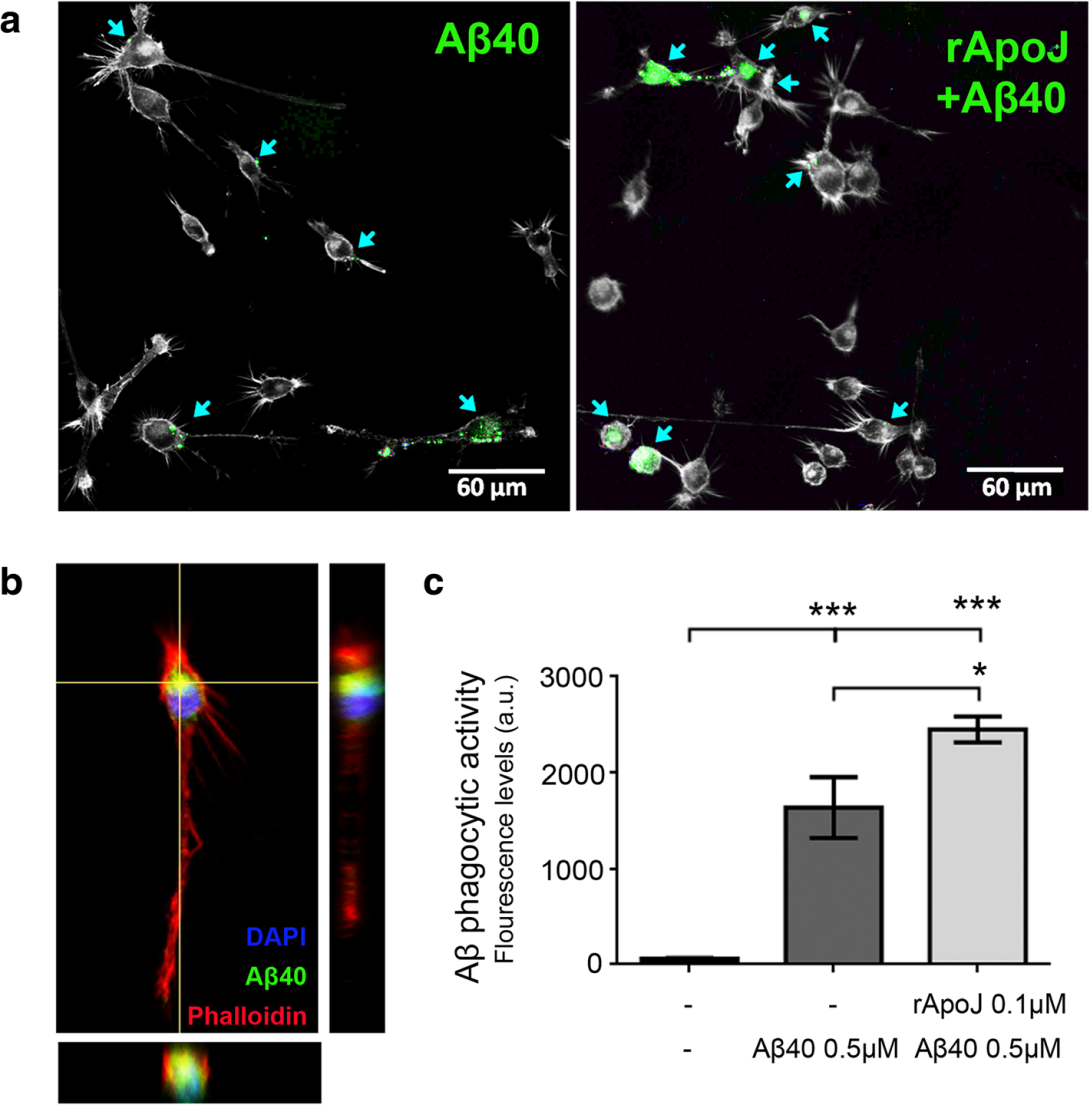
Effect of rApoJ treatment on Aβ phagocytic activity of microglial cultured cells. **a** Representative micrographs corresponding to BV2 cells pre-incubated during 24 h with/without rApoJ (0.1 μM) and then treated for 3 h with HiLyte Fluo 488-Aβ40 (0.5 μM). Blue arrows indicate cells containing HiLyte Fluo 488-Aβ40 (green). Cell morphology, visualized by TRITC-phalloidin staining, and cell nuclei with Dapi are shown in gray. **b** Confocal image showing the z-stack of a single cell containing HiLyte Fluo 488-Aβ40 (green), stained with TRITC-phalloidin (red), and Dapi (blue). **c** Fluorometric quantification of HiLyte Fluo 488-Aβ40 in fixed BV2 cells (Ex/Em = 503/528 nm). Data is expressed as mean ± SEM of 4 independent experiments performed in duplicates. *p < 0.05; ***p < 0.001

Finally, the potential direct effect of free rApoJ on microglial phagocytic activity was tested in vitro. BV2 microglial mouse cells were pretreated with or without rApoJ (0.1 μM) for 24 h and then incubated with HyLite Fluor 488-labeled Aβ_40_ (0.5 μM) for 3 h. Confocal images revealed that rApoJ-pretreated cells retained higher fluorescent-labeled Aβ_40_ levels than non-pretreated cells (Fig. 7a), indicating higher Aβ uptake in cells challenged with rApoJ. The presence of fluorescent-labeled Aβ_40_ inside the cells was corroborated in confocal microscope z-stack images (Fig. 7b). In addition, quantification of HyLite Fluor 488-labeled Aβ_40_ internalized in cultured cells was determined fluorometrically. Results confirmed that rApoJ-pretreated cells significantly enhanced the Aβ phagocytic activity compared with non-pretreated cells (Fig. 7c).

## Discussion

Despite the high socioeconomic impact of AD and CAA, these diseases lack an effective disease-modifying therapy. The involvement of ApoJ in AD is widely acknowledged, although its underlying mechanism in this pathology is not yet elucidated. Therefore, the principal aim of our study was to test the potential protective role of peripheral ApoJ in an experimental model of AD/CAA. To this end, we first considered the need to evaluate the effect of the progressive Aβ deposition on cerebral endogenous mouse ApoJ levels. In fact, we confirmed that, in the APP23 mouse model, the accumulation of both insoluble Aβ_40_ and Aβ_42_ was accompanied by an increase in mouse ApoJ expression in the brain. This increase in ApoJ has also been described in AD and CAA patients [44, 45], although it is not clear whether the increase in brain ApoJ levels promotes or prevents the Aβ accumulation and toxicity. In this regard, the elevated circulating levels of ApoJ observed in AD patients have been proposed to be a protective response against the pathologic Aβ deposition [27]. To confirm this hypothesis, we treated APP23 mice with rApoJ at the time that mouse ApoJ levels elevation was not yet significant. This approach allowed us to study the efficacy of an early increase in peripheral ApoJ in terms of Aβ deposition, neuronal loss, and neuroinflammation. Indeed, the strategy was to increase the circulating levels of ApoJ in mice following two different treatments: intravenous administration of free rApoJ or rHDL-rApoJ nanodiscs. Several lines of evidence support the relevance of cholesterol and HDL metabolism to AD [46], and we previously observed the increased ability of rHDL-rApoJ nanodiscs to promote the efflux of cholesterol esters in vitro [34]. Therefore, we believed that the peripheral administration of rHDL-rApoJ could shed light on the effect of lipidation on the functionality of ApoJ in cerebral β-amyloidosis.

After 1 month of subchronic administration of treatments based on human rApoJ, we confirmed the presence of h-rApoJ in plasma. However, we were not able to detect h-rApoJ in the brains of mice treated with rHDL-rApoJ or free rApoJ. It has previously been described that ApoJ is able to cross the BBB [47]. Based on this, we expected that the infused rApoJ could enter the brain parenchyma, but the human protein was detected by immunofluorescence only on the luminal side of occasional leptomeningeal vessels in both rApoJ-based treatment groups. In fact, we previously observed specific vascular accumulation of rHDL-rApoJ nanodiscs in very old (28 months) APP23 mice [34]. After the corresponding subchronic peripheral treatment in APP23 mice, the cerebral Aβ load was evaluated. Free rApoJ-treated mice showed significant prevention of both insoluble Aβ_40_ and Aβ_42_ accumulation in comparison with the saline-treated group. In turn, the treatment with rHDL-rApoJ nanodiscs resulted in the prevention of insoluble Aβ_42_ accumulation_._ When we analyzed the distribution of Aβ, we observed a reduced number of Aβ-affected vessels in both rApoJ-based treatments. This reduction was in accordance with a previous published study [31], where authors described a marked exacerbation of CAA in APP/PS1/*Clu*^−/−^ mutant mice. The authors proposed that the lack of *Clu* impaired an efficient Aβ clearance across the BBB, promoting the accumulation of Aβ in perivascular spaces. However, this same study showed a clear reduction of parenchymal Aβ accumulation and total Aβ in APP/PS1 mice lacking Clu [31]. After this, another study showed that 5xFAD/*Clu*^−/−^ mice had less Aβ cerebral accumulation only in young mice, although this effect disappeared with age [32]. Beyond the valuable information obtained from these knockout models, we consider that our approach, based on the peripheral administration of rApoJ, allowed us to study the role of human ApoJ in contrast to murine ApoJ and to focus specifically on the effect of a circulating increase in ApoJ levels. As mentioned, our results obtained in aged APP23 mice treated with rApoJ partly agreed with the effect observed in the APP/PS1/*Clu*^*−/−*^ mutant mice described by Wojtas et al. [31], since we detected a significant reduction in the CAA load after treatments. Thus, it can be speculated that the localization of human rApoJ in brain vessels after the peripheral subchronic treatment directly stimulated the clearance of Aβ from perivascular drainage spaces reducing the Aβ vascular load. However, we also observed that the peripheral increase of human rApoJ reduced the total insoluble Aβ in the brain, which was accompanied by a decrease in the size of the fibrillar Aβ deposits. Interestingly, these effects were observed without detecting the human rApoJ protein inside the brain. In fact, intravenous infusion of free ApoJ induced an increase in Aβ levels in CSF, which might reflect an Aβ mobilization process from the brain parenchyma. On the other hand, it has recently been proposed that intraventricular administration of an ApoJ mimetic peptide promotes the amelioration of both parenchymal and vascular Aβ accumulation and improved the cognitive function if the Tg6799 mouse model [48]. These results can be understood as complementary to ours, as they focused only on the intracerebral pool of ApoJ. Therefore, to our knowledge, our study is the first to administrate human recombinant ApoJ, produced in mammalian cells, in a preclinical model of AD. We believe that this is especially relevant due to the high structural complexity of ApoJ and the importance of its full-length form for its functionality [49].

Another beneficial effect of subchronic IV treatment with free rApoJ in APP23 mice was the prevention of neuronal loss in CA1 and poDG of the hippocampus. Due to the association between Aβ deposits and neurodegeneration [50], the reduced Aβ levels observed in free rApoJ-treated mice could explain the amelioration of neuronal death. Once again, as shown by the effect on Aβ load after treatments, rHDL-rApoJ was less effective than the free protein, suggesting that the structural modification of ApoJ due to its lipidation may alter its ability to activate some of the protective pathways within the brain. The inflammatory state of mouse brains after ApoJ-based treatments was also determined. Treatment with free rApoJ or rHDL-ApoJ did not induce changes in the reactivity of astrocytes. Likewise, microglial proliferation was not altered among the treatment groups, but a significant increase in the expression of brain CD68 in free rApoJ-treated mice was observed. CD68 was localized surrounding Aβ parenchymal deposits in cells positive for the TL dye, which stains microglia and other cells, such as macrophages and endothelial cells [40, 51]. In this context, CD68 is a lysosomal protein that has been widely used as a marker of phagocytic activity [52]. Accordingly, we demonstrated that rApoJ had a direct role enhancing the Aβ uptake by microglial cells in vitro, as previously proposed [53]. However, infiltrated monocytes have previously demonstrated a higher ability than microglial cells to phagocytose Aβ peptide [14, 54]. Because resident microglia and peripheral macrophages consist of heterogeneous populations that in many ways are indistinguishable entities [55], the detailed mechanisms implicated in the stimulation of phagocytosis in brain myeloid cells by peripheral ApoJ merit exploration in future studies.

As mentioned, it has been extensively demonstrated that brain myeloid cells participate in Aβ elimination through phagocytosis and that stimulation of the immune response in the CNS ameliorates Aβ deposition [56, 57]. Therefore, promoting the phagocytic ability of these cell types could be a potential strategy to prevent cerebral Aβ deposition. Nevertheless, the exact role of myeloid cells in pathological conditions is under discussion, since it has been observed that, for example, cultured microglia produce high levels of neurotoxic cytokines in response to Aβ stimulation [58]. To test whether the enhancement of phagocytic pathways was accompanied by an increased release of inflammatory molecules, we analyzed the overall inflammatory state of brains after treatments. We did not observe an increase in the cerebral levels of classical proinflammatory molecules after treatments. In fact, reduced neuroinflammation was observed in the brains of rApoJ-treated mice in comparison with saline-treated APP23 mice, as shown by the significant reductions in IL17 and KC (the mouse homolog of CXCL1) levels, especially in free rApoJ-treated mouse brains. In this context, higher expression of IL17 has been linked to BBB disruption and neurotoxicity [59, 60], and KC chemokine levels in the brain have been associated with memory deficits [61]. This last result agrees with previous studies that demonstrated anti-inflammatory and anti-apoptotic properties of ApoJ/Clu [62].

In summary, beyond the accepted implication of ApoJ in cerebral β-amyloidosis, our study is the first to demonstrate that the intravenous administration of human recombinant ApoJ is safe and effective reducing the levels of cerebral insoluble Aβ and the CAA load. Indeed, our results confirm that peripheral interventions offer low invasive opportunities to regulate the cerebral β-amyloid load. An important limitation of our study is the lack of functional analysis after treatments, even though we have demonstrated that free rApoJ treatment prevents neuronal loss in the hippocampus in old APP23 mice. Further studies testing the efficacy of rApoJ treatment using different models of AD will be relevant to analyze its impact on behavior and cognition. We suggest that peripheral rApoJ promotes the amelioration of cerebral β-amyloidosis acting at the vascular level and upregulating specific phagocytic pathways in myeloid cells. However, the exact molecular pathway activated by peripheral ApoJ mediating the stimulation of the phagocytic phenotype needs to be elucidated in future studies.

## Additional file

Additional file 1: **Figure S1.** Effect of rHDL-rApoJ or free rApoJ treatment in the levels of mouse ApoJ in brain homogenates and plasma from treated mice, determined by ELISA. Data are expressed as the mean ± SEM. *N* = 3–4/group. **Figure S2.** Effect of rHDL-rApoJ or free rApoJ treatment in the expression of full-length APP protein and the processing of APP through α-, β-, and γ secretases. a) Representative Western blots showing the relative amount of full-length APP, sAPPα, sAPPβ, and CTF-APP. Relative quantification of b) full-length APP, c) sAPPα, d) sAPPβ, and e) CTF-APP levels after treatments. Data are expressed as the mean ± SEM. *N* = 6/group. **Table S1.** List of abbreviation of tested inflammatory molecules by multiplexed ELISA. (DOCX 302 kb)

## Abbreviations

AD: Alzheimer’s disease
ApoE: Apolipoprotein E
ApoJ: Apolipoprotein J
APP: Amyloid precursor protein
Aβ: β-Amyloid
BBB: Blood-brain barrier
BSA: Bovine serum albumin
CAA: Cerebral amyloid angiopathy
CD68: Cluster of differentiation 68
CHOL: Free cholesterol
Cholate: Sodium deoxycholate
Clu: Clusterin
CNS: Central nervous system
CSF: Cerebrospinal fluid
DAB: Diaminobenzidine
DAPI: 4′,6-Diamidino-2-phenylindole
DMPC: 1,2-Dimyristoyl-sn-glycero-3-phosphocholine
EDTA: Ethylenediaminetetracetic acid
ELISA: Enzyme-linked immunosorbent assay
FBS: Fetal bovine serum
FDR: False discovery rate
FLPC: Fast protein liquid chromatography
free rApoJ: Nonlipidated recombinant ApoJ
GAPDH: Glyceraldehydes 3-phosphate dehydrogenase
GFAP: Glial fibrillary acidic protein
GWAS: Genome-wide association study
HDL: High-density lipoproteins
HEK293T: Human embryonic kidney 293 T cells
h-rApoJ: Human recombinant ApoJ
Iba1: Ionized calcium-binding adapter molecule-1
ICH: Intracerebral hemorrhage
IDE: Insulin-degrading enzyme
IHC: Immunohistochemistry
IL17: Interleukin 17
IL-1β: Interleukin 1-β
IV: Intravenous
KBr: Potassium bromide
KC: Keratinocyte chemoattractant chemokine
LSD: Least significant difference
m-ApoJ: Mouse ApoJ
NEP: Neprilysin
ON: Overnight
poDG: Polymorphic layer of the dentate gyrus
px: Pixel
rHDL-rApoJ: Reconstituted HDL formulated with rApoJ
RT: Room temperature
sAPPα: Soluble APP fraction-α
sAPPβ: Soluble APP fraction-β
TBS: Tris buffer saline
ThS: Thioflavin S
Thy: Thymocyte antigen-1 promoter
TL: Tomato lectin
TNF-α: Tumor necrosis factor-α
WT: Wild-type
