# Supplementary material for: Peripheral administration of human recombinant ApoJ/clusterin modulates brain beta-amyloid levels in APP23 mice
Source: Alzheimers Res Ther. 2019 May 10;11:42. doi: 10.1186/s13195-019-0498-8 (PMC6511153; doi:10.1186/s13195-019-0498-8)
Supplement: Supplementary file 1 — Figure S1. Effect of rHDL-rApoJ or free rApoJ treatment in the levels of mouse ApoJ in brain homogenates and plasma from treated mice, determined by ELISA. Data are expressed as the mean ± SEM. N = 3–4/group. Figure S2. Effect of rHDL-rApoJ or free rApoJ treatment in the expression of full-length APP protein and the processing of APP through α-, β-, and γ secretases. a) Representative Western blots showing the relative amount of full-length APP, sAPPα, sAPPβ, and CTF-APP. Relative quantification of b) full-length APP, c) sAPPα, d) sAPPβ, and e) CTF-APP levels after treatments. Data are expressed as the mean ± SEM. N = 6/group. Table S1. List of abbreviation of tested inflammatory molecules by multiplexed ELISA. (DOCX 302 kb) [file 13195_2019_498_MOESM1_ESM.docx]

**SUPPLEMENTARY INFORMATION**

**
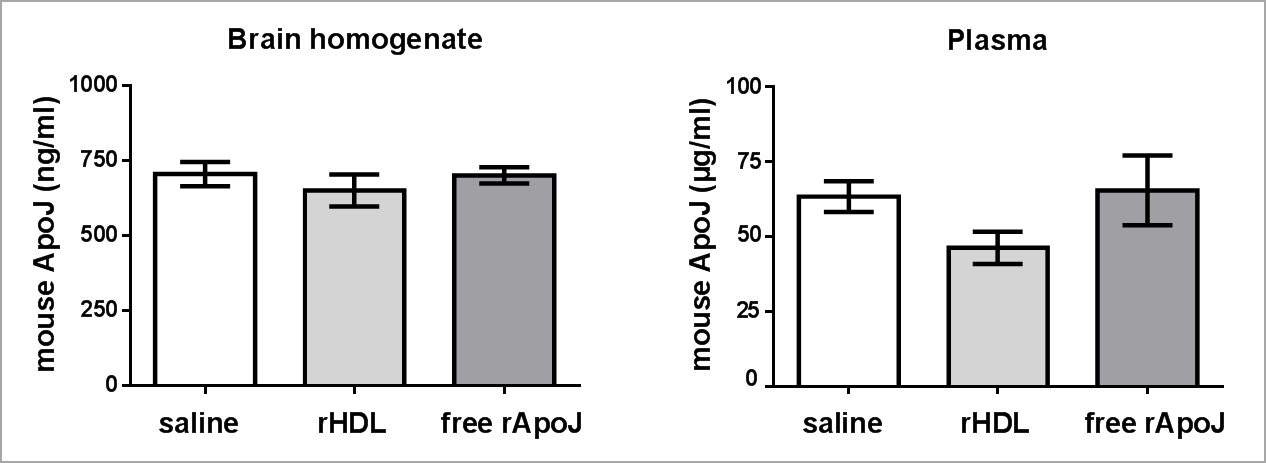
Supplementary Fig. 1**

**Fig. S1:** Effect of rHDL-rApoJ or free rApoJ treatment in the levels of mouse ApoJ in brain homogenates and plasma from treated mice, determined by ELISA. Data are expressed as the mean ± SEM. N=3-4/group.

**Supplementary Fig. 2**


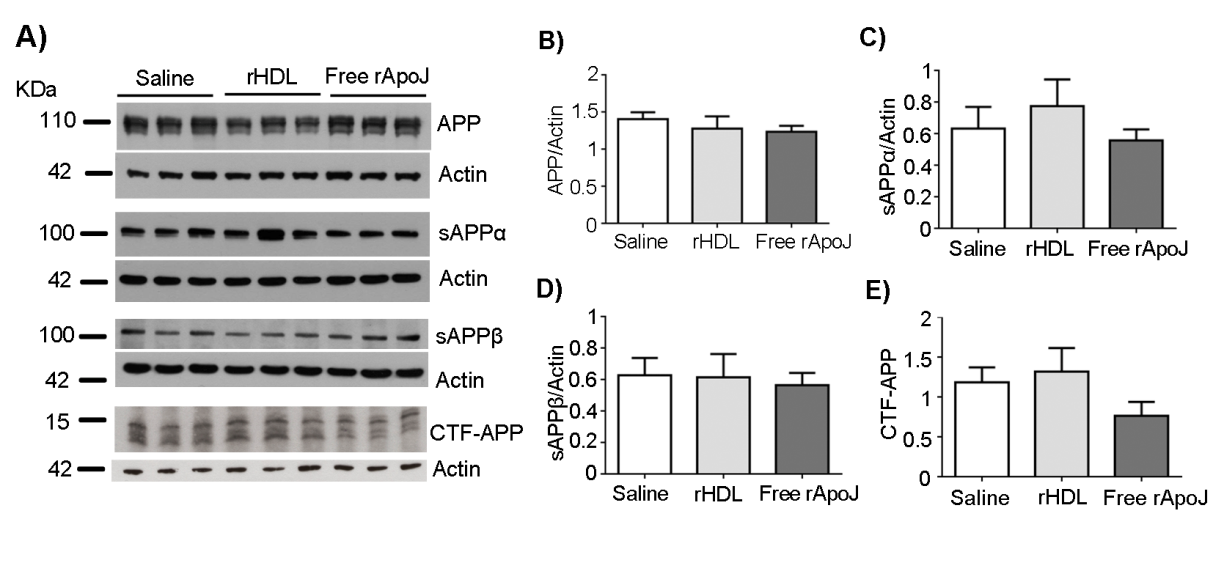


**Fig. S2:** Effect of rHDL-rApoJ or free rApoJ treatment in the expression of full-length APP protein and the processing of APP through α-, β- and γ secretases. a) Representative Western Blots showing the relative amount of full-length APP, sAPPα, sAPPβ and CTF-APP. Relative quantification of b) full-length APP; c) sAPPα; d) sAPPβ; and e) CTF-APP levels after treatments. Data are expressed as the mean ± SEM. N=6/group.

**Supplementary Table 1**

| **Target protein** | **Abbreviation** | **Detection in brain** |
| --- | --- | --- |
| **Basic Fibroblast Growth Factor** | FBF-Basic | Yes |
| **Interleukin-1β** | IL-1β | Yes |
| **Interleukin-10** | IL-10 | No |
| **Interleukin-13** | IL-13 | Yes |
| **Interleukin-6** | IL-6 | Yes |
| **Interleukin-12** | IL-12 | No |
| **Interleukin-17** | IL-17 | Yes |
| **Macrophage Inflammatory Protein-1α** | MIP-1α | No |
| **Granulocyte-Macrophage Colony Stimulating Factor** | GM-CSF | No |
| **Monocyte chemoattractant Protein-1** | MCP-1 | Yes |
| **Interleukin 5** | IL-5 | Yes |
| **Vascular Endothelial Growth Factor** | VEGF | Yes |
| **Interleukin-1α** | IL-1α | No |
| **Interferon-γ** | IFN-γ | Yes |
| **Tumor Necrosis Factor-α** | TNF-α | Yes |
| **Interleukin-2** | IL2 | Yes |
| **Interferon-γ Induced Protein-10** | IP-10 | Yes |
| **Monokine Induced by Gamma-IFN** | MIG | Yes |
| **Keratinocyte Chemoattractant** | KC (mouse CXCL1) | Yes |
| **Interleukin-4** | IL-4 | No |

**Table S1:** List of abbreviation of tested inflammatory molecules by multiplexed ELISA.
